# *QuickStats:* Percentage* of Adults Who Received Any Mental Health Treatment in the Past 12 Months,^†^ by Age Group and Year — National Health Interview Survey, United States, 2019–2020^§^

**DOI:** 10.15585/mmwr.mm7043a5

**Published:** 2021-10-29

**Figure:**
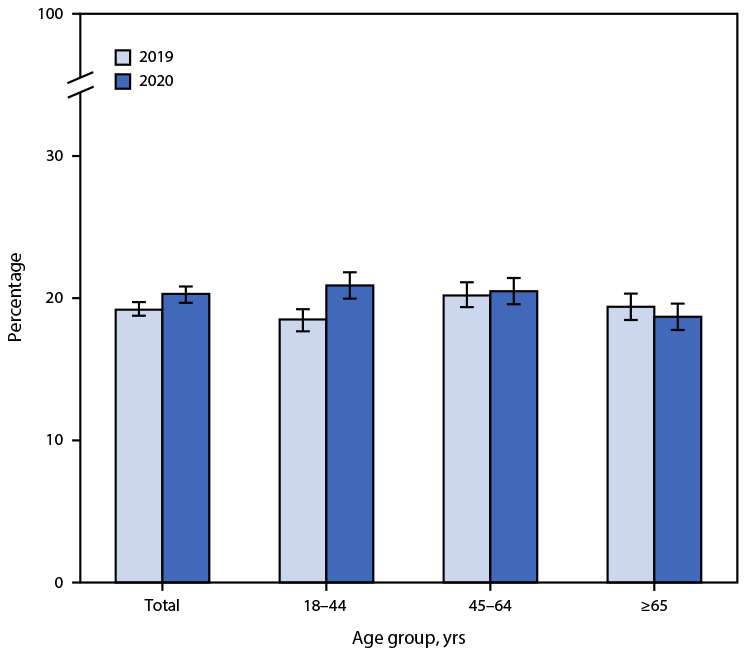
The percentage of adults who had received any mental health treatment in the past 12 months increased from 2019 to 2020 overall (19.2% to 20.3%) and among adults aged 18–44 years (18.5% to 20.9%). In 2019, the percentage of adults who had received any mental health treatment in the past 12 months was lower among those aged 18–44 years (18.5%) compared with those aged 45–64 years (20.2%) and ≥65 years (19.4%). In 2020, the percentage decreased with age, from 20.9% among adults aged 18–44 years to 18.7% among those aged ≥65 years.

**Sources:** National Center for Health Statistics. NCHS data brief, no. 380. https://www.cdc.gov/nchs/data/databriefs/db380-H.pdf; NCHS data brief, no. 419. https://www.cdc.gov/nchs/data/databriefs/db419.pdf

